# Serum levels of miR-223 but not miR-21 are decreased in patients with neuroendocrine tumors

**DOI:** 10.1371/journal.pone.0244504

**Published:** 2020-12-31

**Authors:** Teresa Hellberg, Raphael Mohr, Lukas Geisler, Jana Knorr, Alexander Wree, Münevver Demir, Fabian Benz, Joeri Lambrecht, Sven H. Loosen, Frank Tacke, Christoph Roderburg, Henning Jann, Burcin Özdirik

**Affiliations:** 1 Department of Hepatology & Gastroenterology, Charité University Medicine Berlin, Berlin, Germany; 2 Medical Faculty of Heinrich Heine University Düsseldorf, Clinic for Gastroenterology, Hepatology and Infectious Diseases, University Hospital Düsseldorf, Düsseldorf, Germany; Institut de Pharmacologie Moleculaire et Cellulaire, FRANCE

## Abstract

**Background and aims:**

MicroRNAs (miRNAs) are profoundly involved into the pathophysiology of manifold cancers. Recent data suggested a pivotal role of miRNAs as biomarkers in different biological processes including carcinogenesis. However, their role in neuroendocrine tumors (NETs) is only poorly understood.

**Methods:**

We determined circulating levels of miR-21 and miR-223 in 45 samples from patients with NET treated between 2010 and 2019 at our department and compared them to healthy controls. Results were correlated with clinical records.

**Results:**

In the total cohort of Patients with NET, miR-223 presented significantly lower levels compared to healthy control samples. In contrast, levels of miR-21 indicated no significant changes between the two groups. Interestingly, despite being significantly downregulated in all NET patients, concentrations of miR-223 were independent of clinical or histopathological factors such as proliferation activity according to Ki-67 index, tumor grading, TNM stage, somatostatin receptor expression, presence of functional/ non-functional disease or tumor relapse. Moreover, in contrast to data from recent publications analyzing other tumor entities, levels of miR-223 serum levels did not reflect prognosis of patients with NET.

**Conclusion:**

Lower concentrations of circulating miR-223 rather reflect the presence of NET itself than certain tumor characteristics. The value of miR-223 as a biomarker in NET might be limited to diagnostic, but not prognostic purposes.

## Introduction

Diagnosis of neuroendocrine tumors (NET) mainly relies on histopathological analyses. In contrast to other tumor entities where a set of biomarkers (“liquid biopsy”) have been proposed as an alternative to histology, no serum based markers do exist so far for a reliable detection of NET [1]. Chromogranin A (CgA) represents the most prominent marker in the context of NET. However, it is rather used for monitoring tumor response on treatment, than for the initial diagnostic process of NET [2,3]. The lack of easily accessible biomarkers represents a major drawback in early diagnosis of NET and many patients are first diagnosed in advanced disease stages lacking curative treatment options. Therefore, innovative parameters reflecting novel pathophysiological concepts are eagerly needed to improve the clinical management of patients with NET [4].

MicroRNAs (miRNAs) are small noncoding RNAs that are known to negatively regulate the expression of their target genes on a posttranscriptional and posttranslational level [5]. About 50% of the human transcriptome is controlled by miRNAs. For inhibition of gene expression perfect base pairing between miRNA and the respective target messenger RNA (mRNA) does not represent an essential prerequisite. Therefore, one miRNA might regulate dozens of mRNAs and, in turn, one mRNA might be regulated by different miRNAs [6,7]. Specifically in carcinogenesis, alterations in miRNA expression levels have been demonstrated in almost all tumor entities highlighting the deep integration of miRNAs in the pathophysiology of cancers [8–10]. Furthermore, miRNAs may act as tumor promotor (oncomiRs) or as tumor suppressors by targeting genes involved in proliferation, cell cycle control, apoptosis, invasion, and drug resistance [11].

miRNAs were suggested as next generation biomarkers as they bear some important advantages over classical protein-based biomarkers. First the number of miRNAs is much smaller than that of proteins, second their chemical complexity is considerably lower and third they remain stable even under conditions that would lead to degradation of most proteins. Notably, the specific source of circulating miRNAs has not yet been defined and might even be disease specific [12].

Recent evidence suggest that miRNAs are involved in the specific carcinogenesis of NET. For example, a pilot study demonstrated that four serum miRNAs (miR-125b-5p, -362-5p, -425-5p and -500a-5p) are up-regulated in small bowel NET [13]. Furthermore, a dysregulation of the proliferation-related miRNA miR-21 and the immune-related miRNA miR-223 were associated with gastroenteropancreatic NET [14–16].

We hypothesized that levels of both, miR-21 and miR-223, undergo deregulation and therefore represent biologically plausible markers in the context of NET. In the present study, we measured serum levels of miR-21 and miR-223 in 45 patients with NET, compared them to healthy controls and correlated results to patients’ clinical course.

## Materials and methods

### Design of study and patient cohort

In this study, we evaluated circulating levels of miRNAs in a cohort of 45 patients with NET, which were treated at Charité - Universitätsmedizin Berlin, a tertiary health care center that provides advanced specialty care to patients with NEN. Our NEN database comprises 612 patients with histologically proven diagnosis of NEN from January 2010 to August 2019. We could identify 45 patients, that were recruited between January 2000 and October 2013, fulfilling our inclusion criteria. Our inclusion criteria comprised i) histopathologically proven diagnosis with NET according to WHO classification 2010 after biopsy or tumor resection [17], (ii) gastroenteropancreatic origin and (iii) serum samples collected between January 2000 and October 2013. Our exclusion criteria comprised (i) a non-gastroenteropancreatic origin and (ii) a diagnosis with neuroendocrine carcinoma according to WHO classification 2010 after biopsy or tumor resection [17]. Our patient cohort was randomly selected from the existing database and can therefore be considered representative of a larger population. Patients were recruited during regular presentations at our university hospital. Patients’ blood samples were collected and were centrifuged for 10 min at 2000 g. In order to avoid repetitive freeze-thaw cycles until use serum aliquots of 1 ml were frozen immediately at -80°C. 19 healthy blood donors, who showed no evidence of a malignant tumor, served as control samples. Patients were included into the study upon providing written informed consent. The study protocol was approved by the Ethics committee of Charité, University Medicine Berlin, Germany (ethical approval number EA1/229/17).

### miRNA isolation from serum

Total RNA was isolated from human serum samples using the miRNeasy Serum/Plasma Advanced Kit (Qiagen, Hilden, Germany) according to the manufacturer’s instruction. Afterwards 300 μl serum was transferred into a 2 ml microcentrifuge tube, 90 μl buffer RPL (Qiagen, Hilden, Germany), which contains guanidine thiocyanate as well as detergents, was added, vortexed and incubated at room temperature (RT) for 3 min. To precipitate inhibitors (mostly proteins that are highly concentrated in serum samples), 90 μl buffer RPP was added, mixed vigorously followed by an incubation of 3 min at RT. Samples were centrifuged for 3 min at 12000 x *g* (Eppendorf Centrifuge 5415 R, Hamburg, Germany) at RT until complete phase separation. The aqueous phase, containing total RNA, was precipitated with one volume (350–375 μl) 100% isopropanol. In a next step, the entire sample was transferred to a RNeasy UCP MinElute column and centrifuged for 15 s at 8000 x *g*. 700 μl buffer RWT (Qiagen, Hilden, Germany) was added, followed by centrifugation for 15 s at 8000 x *g* RT. Afterwards, 500 μl 80% ethanol were added, followed by centrifugation at RT for 15 s at 8000 x *g*. Total RNA was eluted with 20 μl RNase-free water and stored at −80 °C.

### Quantitative real-time PCR

Quantitative real-time polymerase chain reaction (qPCR) was performed as recently described [5,18]. In brief, 5 μl of extracted total RNA was used to synthesize complementary deoxyribonucleic acid (cDNA) utilizing miScript Reverse Transcriptase Kit (Qiagen, Hilden, Germany) according to the manufacturer’s protocol. cDNA samples (2 μl) were used for quantitative real-time PCR (Applied Biosystems 7300 Sequence Detection System, Applied Biosystems, Foster City, CA) in a total volume of 25 μl using the miScript SYBR Green PCR Kit (Qiagen, Hilden, Germany) and the miRNA specific primers miR-21 (hsa-miR-21-5p; MIMAT0000076: 5'-UAGCUUAUCAGACUGAUGUUGA-3'), miR-223 (hsa-miR-223-3p; MIMAT0000280: 5-'UGUCAGUUUGUCAAAUACCCCA-3') as well as miR-16 (hsa-miR-16-1-3p; MIMAT0004489: 5'-CCAGUAUUAACUGUGCUGCUGA-3') for data normalization (Qiagen, primer sequences available online). All results are given as 2-ΔΔCT and represent the x-fold increase of gene expression in relation to our housekeeping gene miR-16. Data were generated and analyzed using the SDS 2.3 and RQ manager 1.2 software packages.

### Statistical analysis

Serum data are displayed as scatter plots. Non-parametric data were compared using the Mann-Whitney *U* test or the Kruskal-Wallis-Test for multiple group comparisons. Correlation analyses were performed using the Spearman's correlation coefficient. Scatter plots display the ranges. We generated receiver operating characteristic (ROC) curves by plotting the sensitivity (%) against 100%—specificity (%). Kaplan-Meier curves display the impact of a specific parameter on the overall survival. The respective 95% confidence intervals were estimated with the Kaplan–Meier survival method. Survival curves between groups were compared by the Log-rank Mantel-Cox test. Given the exploratory character of this study, we refrained from sample size and power calculations. All statistical analyses were performed with Prism (version 7.03; GraphPad, La Jolla, California, USA). A *p* value of <0.05 was considered statistically significant (*p <0.05; **p <0.01; ***p <0.001).

## Results

### Patient characteristics

In the present analysis 45 patients with histologically confirmed NET were included. Out of these, 24 (53%) were female. Median age at initial diagnosis was 59 years (17–80 years). Primary tumor localizations were ileum (n = 23) and pancreas (n = 21) as well as the stomach (n = 1). Median time of follow-up was 9 years (range 0–21 years), and the median Ki-67 proliferation index was 2% (range 1–50%). 23 (52%) tumors were histologically characterized as Grade 1, 17 (39%) as Grade 2, and 4 (9%) as Grade 3. Patient and tumor characteristics are given in Table 1.

**Table 1:** Patient and tumor characteristics.

Characteristics	All patients n = 45 (100%)
Sex, female	24 (53%)
Age at initial diagnosis, median	59 (17–80)
Patient age • ≤ 65 • > 65	• 28 (62%) • 17 (38%)
Comorbidities • Diabetes • Arterial hypertension	• 7/27 (26%) • 13/27 (48%)
Primary tumor localization • ileum • pancreas • stomach	• 23 (51%) • 21 (46%) • 1 (2%)
• Median survival (years) • Median survival (months) • No. of patients alive • No. of death patients • No. of patients lost-to-follow-up	9 (0–21) 112 (0–247) • 16 (36%) • 11 (24%) • 18 (40%)
Treatment at time of serum sampling • No treatment • Surgical resection of primary tumor and/or metastases • Biological therapy (SSA, IFN) • Radiation therapy • PRRT	• n = 8 (18%) • n = 30 (67%) • n = 11 (24%) • n = 1 (2%) • n = 1 (2%) • n = 1 (2%)
Treatment during course of disease • Surgical resection of primary tumor and/or metastases • Treatment with biological therapy (SSA, IFN) • Treatment with chemotherapy • Treatment with PRRT	• n = 42 (93%) • n = 18 (40%) • n = 3 (7%) • n = 3 (7%) • n = 16 (16%)
Ki-67 (Median; range) • Ki-67 ≤ 3 • Ki-67 > 3 and ≤ 10 • Ki-67 > 10 and ≤ 20	(2%; 1%–50%) • 26/43 (60%) • 14/43 (33%) • 3/43 (7%)
T stage • T1 • T2 • T3 • T4	n = 35 • 2/35 (6%) • 12/35 (34%) • 15/35 (43%) • 6/35 (17%)
Lymph node metastases • no • yes	• 9/44 (20%) • 35/44 (80%)
Metastases • No • Yes	• 11/44 (25%) • 34/44 (75%)
Hepatic metastases • No • Yes	• 16/44 (36%) • 28/44 (64%)
Peritoneal carcinomatosis • No • Yes	• 33/44 (75%) • 11/44 (25%)
Relapse • No • Yes	• 30/42 (71%) • 12/42 (29%)
Functional disease • No • Yes	• 23/41 (56%) • 19/41 (46%)
SSR expression • No • Yes	• 6/41 (15%) • 35/41 (85%)

Data are n (%) of patients, if not indicated otherwise. The percentages were rounded and may not sum 100%. Where the denominator is shown, data were not available for all patients.

### Circulating levels of miR-223 are lower in NET patients

**Fig 1:**
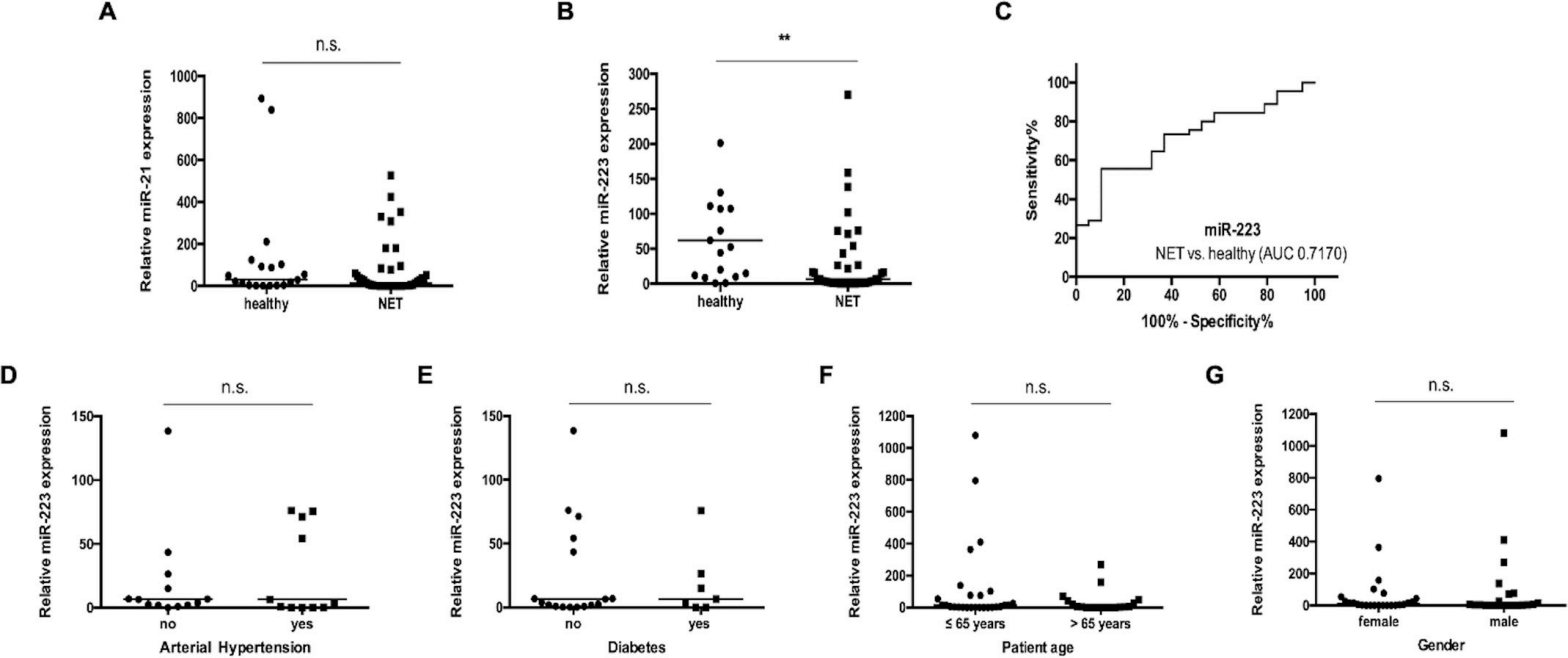
Circulating levels of miR-223 are lower in NET patients. (**A**) Analysis of circulating miR-21 concentrations does not show a significant correlation between neuroendocrine tumor (NET) patients and healthy controls. (**B**) In contrast, circulating levels of miR-223 are significantly lower in patients with NET compared to healthy controls. (**C**) Receiver operating characteristic (ROC) curve analysis of miR-223 levels reveals an area under the curve (AUC) value of 0.7170 regarding the discrimination of NET patients and healthy controls. There is no significant correlation between miR-223 concentrations in serum and the presence of (**D**) arterial hypertension (**E**) type 2 diabetes (**F**) age (younger/ older than 65 years) and (**G**) gender. The scatter plots display relative miR-223 and miR-21 expression levels between two subgroups. The black horizontal lines represent the median per group. (** p < 0.01). Neuroendocrine tumors (NET).

miRNAs are deeply involved into the pathophysiology NET [14–16,19–21]. Based on these data and the suggested role of miR-21 and miR-223 in manifold cancers [22–27], we analysed the potential role of these miRNAs as serum-based markers in patients with NET. Therefore, we measured concentrations of circulating miR-21 and miR-223 in 45 patients with NET and 19 healthy blood donors as controls. Interestingly, while levels of miR-21 (Fig 1A) were similar between NET-patients and the control group, concentrations of miR-223 were significantly lower in NET-patients compared to the healthy controls (Fig 1B). To quantify the discriminatory power of miR-223 for distinguishing between NET and controls we next applied receiver operating characteristic (ROC) curve analyses revealing an area under the curve (AUC) of 0.7170 for discriminating between patients and healthy controls without any type of cancer (Fig 1C). Of note, at the ideal cut-off value of 7.750 [AU], the sensitivity for diagnosis of NET was 89.47% with a specificity of 55.56%.

Levels of miR-223 were recently described in patients with cardiovascular and metabolic diseases [28,29]. Therefore, we subdivided our cohort of patients in those with cardiovascular and metabolic diseases and those without, respectively, and compared miR-223 concentrations. Notably, in our cohort, miR-223 concentrations were independent of the presence of these comorbidities (Fig 1D and 1E) and not associated to the patient’s age or sex (Fig 1F and 1G).

### miR-223 levels do not reflect disease characteristics in patients with NET

**Fig 2:**
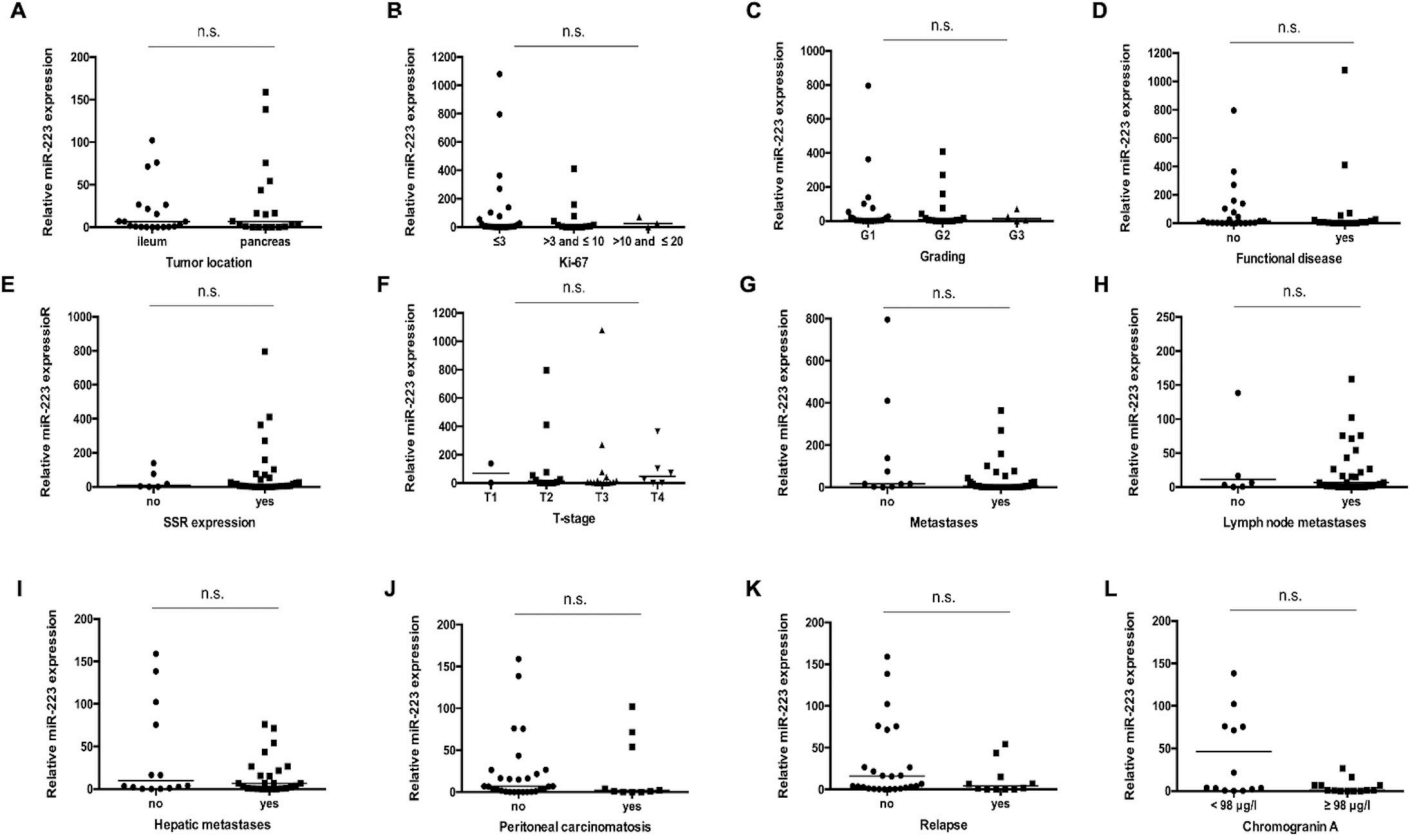
miR-223 levels do not reflect disease characteristics in patients with NET. There is no significant correlation between miR-223 concentration levels in serum of neuroendocrine tumor patients with respect to (**A**) different tumor localization, (**B**) different Ki-67 rates, (**C**) different histological tumor grading (Grade 1 to 3), the presence of (**D**) functional or non-functional disease and (**E**) SSR positive or negative disease. Analysis of different subgroups with (**F**) different T-stages, (**G**) non-metastasized and metastasized disease, (**H**) lymph node positive or negative disease, (**I**) with/ without hepatic metastases and (**J**) with/ without peritoneal carcinomatosis does not reveal a significant difference. Moreover, (**K**) a positive or negative postoperative relapse status and (**L**) higher or lower Chromogranin A levels (cut-off value of 98 μg/l (median)) in NET patients do not show any significant correlation. The scatter plots display relative miR-223 expression levels between different subgroups. The black horizontal lines represent the median per group. (* p < 0.05; *** p < 0.001).

Based on the downregulation of miR-223 in patients with neuroendocrine tumors we hypothesized that levels of circulating miR-223 might be indicative for specific clinicopathological characteristics. We therefore analyzed miR-223 levels with respect to different tumor localizations (Fig 2A), different Ki-67 rates (Fig 2B), different histological tumor grading (Fig 2C), presence of functional or non-functional disease (Fig 2D) as well as positive or negative somatostatin receptor (SSR) expression status (Fig 2E). Furthermore, we analyzed miR-223 concentrations in patients with advanced or earlier disease (Fig 2F), presence of metastases (Fig 2G), lymph node positive or negative disease (Fig 2H) as well as in patients with or without hepatic metastases (Fig 2I) and peritoneal carcinomatosis (Fig 2J). However, no significant differences became apparent between the different groups, which might be due to the small sample size used in this study. Finally, we compared miR-223 concentrations between patients that displayed a tumor relapse after surgery with those in patients with sustained tumor response. This analysis also did not reveal differences in miR-223 concentrations between the NET- and healthy group (Fig 2K). We next hypothesized that patients with a higher tumor load according to elevated Chromogranin A (CgA) concentrations might display further decrease in miR-223 concentrations. However, when a cut-off value of 98 μg/l (median) was used, patients with higher CgA concentrations displayed similar miR-223 levels compared to patients with lower CgA concentrations (Fig 2L).

**Fig 3:**
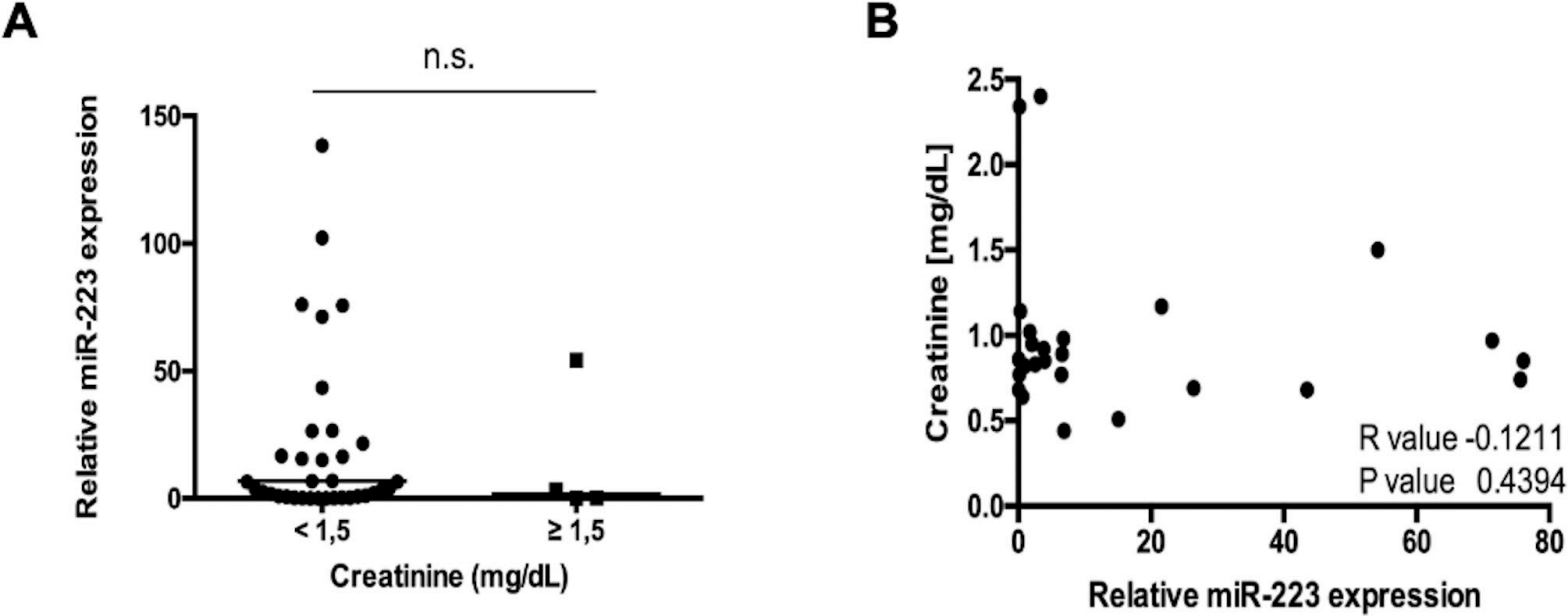
No significant correlation between miR-223 and impaired kidney function. (**A**). Serum levels of miR-223 in patients with NET in correlation to serum creatinine levels. The scatter plot displays relative miR-223 expression levels between two subgroups. The black horizontal line represents the median per group. (* p < 0.05; *** p < 0.001). (**B**) In line, Spearman rank analysis does not reveal a significant correlation between creatinine values and relative miR-223 levels of NET patients (R = -0.1211, p = 0.4394).

Recently, a direct link between miRNA serum concentrations and an impaired kidney function was suggested since circulating miRNAs are cleared by the kidney. We therefore specifically analyzed miR-223 levels in patients with creatinine concentrations ≥ 1.5 mg/dl and those with lower levels. Interestingly, both groups displayed similar miR-223 concentrations (Fig 3A). In line, spearman rank analysis did not reveal a correlation between miR-223 and creatinine concentrations (Fig 3B).

### Circulating miR-223 levels do not reflect overall survival in patients with NET

**Fig 4:**
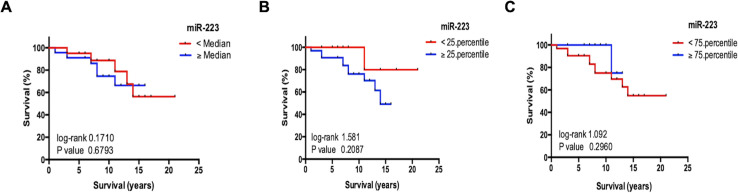
Circulating miR-223 levels do not reflect overall survival in patients with NET. Kaplan-Meier analysis of miR-223 levels in serum of NET patients above (red curve) and below (blue curve) (**A**) the median (6.61 [AU]), (**B**) the 25^th^ percentile (0.74 [AU]) and (**C**) 75^th^ percentile (62.75 [AU]) displays a similar outcome. There is no significant correlation between levels of circulating miR-223 and the patient’s survival time. (* p < 0.05; *** p < 0.001).

In a next step, we analyzed if the miR-223 serum levels could reflect the overall prognosis of the patients. Therefore, we used Kaplan Meier curve analysis revealing that patients with miR-223 concentrations higher or lower than the median, 25^th^, or 75^th^ percentile of all patients displayed an almost identical outcome (Fig 4A–4C). Additionally, we found no correlation between levels of circulating miR-223 and the patient’s survival time. Thus, we concluded that circulating miR-223 has no immediate value for prognosis prediction in NET.

### Circulating levels of miR-21 do not show a significant correlation in respect to patient characteristics, disease characteristics and overall survival

In line with miR-21 levels not showing any significant difference between NET patients and the control group, we could not detect a significant correlation between miR-21 levels in serum of NET patients and patient characteristics such as the presence of comorbidities, age and gender (S1 Fig). Furthermore, our analysis revealed no association of miR-21 levels in serum in NET patients with disease characteristics such as tumor localization, Ki-67, histological grade, functional disease, SSR positivity, different T-stages, presence of metastases, relapse status, CgA levels (S2 Fig). Moreover, miR-21 levels were not associated with overall survival in NET patients (S3 Fig).

## Discussion

Our present data corroborate the hypothesis that serum concentrations of miR-223 but not of miR-21 might be altered in patients with neuroendocrine tumors. Nevertheless, in this rather small cohort no correlation between miRNA serum levels and patient or tumor specific characteristics could be found.

We analyzed two biologically plausible miRNAs as potential serum biomarkers in patients with NET. miR-21 is a well-known player in carcinogenesis, being frequently overexpressed in various human tumors and in cancer cell lines. Its expression is regulated by STAT3 and NF-κB transcription factors, which are both activated in a variety of cancers playing critical roles in the regulation of cell proliferation, invasion, apoptosis, and tumorigenesis [30]. Dysregulation of miR-21 levels are described for almost all types of gastrointestinal cancers [31–33].

The second possible miRNA miR-223 functioned in mouse models as a negative regulator of DNA repair mechanisms [33]. Furthermore, there is recent evidence that miR-223 expression is related to lymphovascular invasion and distant metastasis in bladder cancer [24]. In line, it was demonstrated that downregulation of miR-223 contributes to an epithelial-mesenchymal transition and thus promotes metastasis in gastric cancer [34].

In our cohort of patients with NET, miR-223 was significantly down-regulated when compared with healthy controls. Nevertheless, in our analysis miR-223 did not correlate with patients or tumor specific factors and was linked neither to the tumor grading nor to the tumor proliferation activity. These explorative data might indicate that lower concentrations of circulating miR-223 rather reflect the presence of NET itself than certain tumor characteristics. Thus, it might be hypothesized that miR-223 is more likely originating from immune cells than from the actual tumor cells. miRNAs were shown to be key regulators of the crosstalk between cancer cells and immune cells. Innate immune cells, particularly macrophages and granulocytes, regulate via miRNAs the immune development of a tumor’s microenvironment [35].

Moreover, these data raise the questions, which cells represent the origin of circulating miR-21/miR-223 in patients with NET and whether miRNAs are passively released into the blood circulation or released in an actively regulated process. The fact that concentrations of circulating miRNA do not correlate with tumor or patient specific factors suggest a passive release. However, the data presented here do not allow to finally answer these questions. Recently, it was suggested that circulating exosomal miRNAs can regulate gene expression in distant tissues and have far-reaching systemic effects [36,37]. As an example, Liu et al. demonstrated that cancer cells-derived exosomal miRNAs can reduce the resistance of ovarian cancer cells to platinum derived chemotherapy. miR-223 plays a pivotal role in the regulation of immune cells and systemic inflammatory processes. Thus, tumor derived miR-223 might directly influence gene expression networks that lead to the activation of immune cells and create a pro- or anti-inflammatory microenvironment potentially allowing tumors to escape immune surveillance. Corroborating this hypothesis, tumor-associated myeloid-derived suppressor cells (MDSCs), including mono- and polynuclear subsets, were demonstrated to express lower miR-223 when compared with CD11b^+^Gr1^+^ cells from cancer-free mice [38].

Based on miRNAs’ tissue-specific expression, their rapid release into the blood flow and their stability in plasma, circulating miRNAs are presently scrutinized for their capability as biomarkers for NET both in a diagnostic and prognostic setting [39]. Measurements of circulating miRNAs might serve as a potential new approach for prompt and non-invasive diagnostic / prognostic screening using real-time PCR. Our data support the use of serum levels of miR-223 as a novel tool for diagnosis of NET. miR-223 might be of particular value when integrated into a panel of other parameters rather than when being used as a single marker.

Our study bears some important limitations. Sample size is rather small as patients with NET are scarce. This fact also accounts to the heterogenicity of our cohort in terms of different tumor grading and localization included. Moreover, the small patient numbers included into different subgroup analysis might have masked existing differences between these groups. Since all patients were treated at a single center, confirming the results in a multi-center study would greatly strengthen the data. In addition, our study did not include longitudinal measurements during treatment, such as chemotherapy or loco-regional therapies, and we cannot provide data showing whether the course of circulating miR-223 reflects tumor response or whether a further decrease in miR-223 concentrations might have a different outcome than in patients whose levels increase. Furthermore, there is no consensus about standard controls for the normalization of circulating miRNA levels in blood samples especially in the exceedingly rare cohort of patients with NET. Quite different strategies including the use of endogenous and exogenous controls/ normalizers in the context of circulating miRNA exist, but all are controversially discussed in many aspects. Under these circumstances, an endogenous miRNA miR-16 was chosen for normalization of circulating miRNAs based on recent studies [40–47]. In summary, this exploratory analysis should trigger further research on the important question whether non-coding RNA and, in particular miRNA, are involved in the complex pathophysiology of NET and might be used as diagnostic or prognostic markers in patients.

## Supporting information

S1 Fig Circulating miR-21 levels do not show a significant correlation in respect to patient characteristics. Concentrations of miR-21 in serum are independent of the presence of (**A**) arterial hypertension (**B**) type 2 diabetes (**C**) age (younger/ older than 65 years) and (**D**) gender. The scatter plots display relative miR-21 expression levels between two subgroups. The black horizontal lines represent the median per group. (* p < 0.05; *** p < 0.001).
(TIF)

S2 Fig Circulating levels of miR-21 do not show a significant correlation with regard to disease characteristics. There is no significant correlation between relative miR-21 concentration levels in serum of NET patients with respect to (**A**) tumor localization, (**B**) Ki-67 rates, (**C**) histological tumor grading (Grade 1 to 3), the presence of **(D)** functional or non-functional disease and (**E**) SSR positive or negative disease. Analysis of the subgroups with (**F**) different T-stages, (**G**) presence of metastases, (**H**) lymph node positive or negative disease, (**I**) with/ without hepatic metastases and (**J**) with/ without peritoneal carcinomatosis does not reveal any significant difference. Moreover, (**K**) a positive or negative postoperative relapse status and **(L)** higher or lower Chromogranin A levels (cut-off value of 98 μg/l (median)) in NET patients do not show any significant correlation. The scatter plots display relative miR-21 expression levels between different subgroups. The black horizontal lines represent the median per group. (* p < 0.05; *** p < 0.001).
(TIF)

S3 Fig Circulating miR-21 levels do not reflect overall survival in patients with NET. sCorrelation between levels of circulating miR-21 and the patient’s survival time is no significant (* p < 0.05; *** p < 0.001).
(TIF)

## Abbreviations

AU: Arbitrary units
AUC: Area under the curve
CCA: Cholangiocellular carcinoma
CgA: Chromogranin A
cDNA: complementary deoxyribonucleic acid
mRNA: messenger RNA
miRNAs: MicroRNAs
MDSC: Myeloid-derived suppressor cell
NEC: Neuroendocrine carcinoma
NET: Neuroendocrine tumor
PCR: Polymerase chain reaction
RT: Room temperature
ROC: Receiver operating characteristic
SSR: Somatostatin receptor
