# Supplementary figures and images for: Serum levels of miR-223 but not miR-21 are decreased in patients with neuroendocrine tumors
Source: PLoS One. 2020 Dec 31;15(12):e0244504. doi: 10.1371/journal.pone.0244504 (PMC7775044; doi:10.1371/journal.pone.0244504)

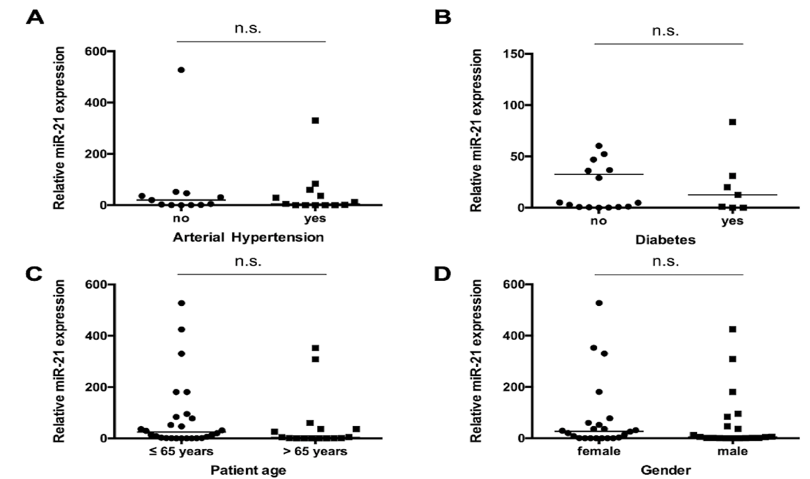

Supplement: S1 Fig — Concentrations of miR-21 in serum are independent of the presence of (A) arterial hypertension (B) type 2 diabetes (C) age (younger/ older than 65 years) and (D) gender. The scatter plots display relative miR-21 expression levels between two subgroups. The black horizontal lines represent the median per group. (* p < 0.05; *** p < 0.001). (TIF) [file pone.0244504.s001.tif]

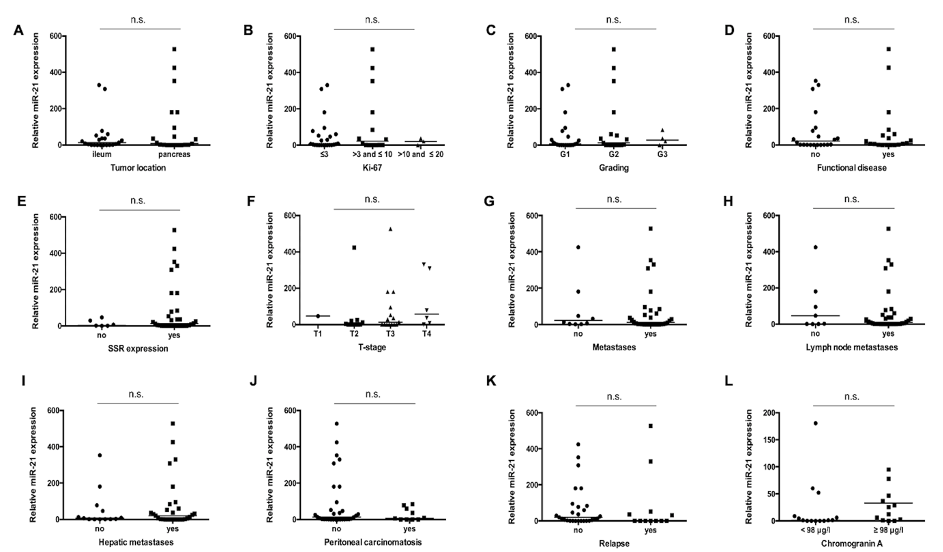

Supplement: S2 Fig — There is no significant correlation between relative miR-21 concentration levels in serum of NET patients with respect to (A) tumor localization, (B) Ki-67 rates, (C) histological tumor grading (Grade 1 to 3), the presence of (D) functional or non-functional disease and (E) SSR positive or negative disease. Analysis of the subgroups with (F) different T-stages, (G) presence of metastases, (H) lymph node positive or negative disease, (I) with/ without hepatic metastases and (J) with/ without peritoneal carcinomatosis does not reveal any significant difference. Moreover, (K) a positive or negative postoperative relapse status and (L) higher or lower Chromogranin A levels (cut-off value of 98 μg/l (median)) in NET patients do not show any significant correlation. The scatter plots display relative miR-21 expression levels between different subgroups. The black horizontal lines represent the median per group. (* p < 0.05; *** p < 0.001). (TIF) [file pone.0244504.s002.tif]

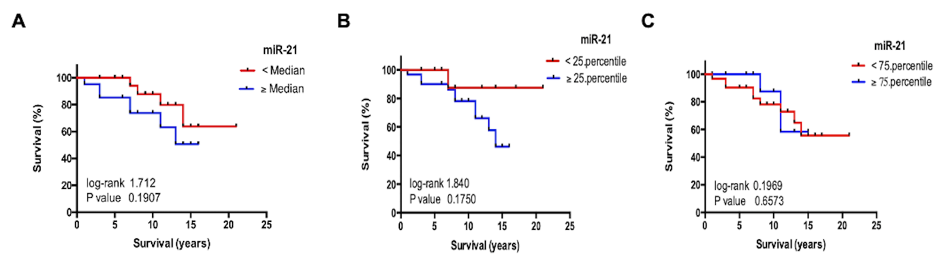

Supplement: S3 Fig — sCorrelation between levels of circulating miR-21 and the patient’s survival time is no significant (* p < 0.05; *** p < 0.001). (TIF) [file pone.0244504.s003.tif]
